# Delayed transient obstructive hydrocephalus after cerebral aneurysm rupture: A case report

**DOI:** 10.1097/MD.0000000000026228

**Published:** 2021-06-04

**Authors:** Yuanhong Ge, Qingjia Lai, Wenyu Wang, Xuejun Xu

**Affiliations:** a Department of Neurosurgery, Chengdu Second People's Hospital; b Department of Rehabilitation, The Second affiliated Hospital of Chengdu Medical College & Nuclear Industry 416 Hospital, Chengdu, China.

**Keywords:** cerebral aneurysm, intraventricular hemorrhage, obstructive hydrocephalus

## Abstract

**Rationale:**

Obstructive hydrocephalus (OH) frequently occurs in patients with a ruptured cerebral aneurysm (CA), and it may lead to severe neurological deficits, including life-threatening brain herniation. OH generally occurs in the early stage of CA rupture, rather than in the late stage, and rarely resolves without therapy.

**Patient concerns:**

A 64-year-old woman with a ruptured anterior communicating artery aneurysm was treated with coil embolization. Nineteen days after her CA rupture, because of the delayed transient OH, she experienced a dramatic cycle in consciousness over 9 hours: wakefulness–drowsiness–coma–drowsiness–wakefulness.

**Diagnosis:**

The patient was diagnosed with delayed transient obstructive hydrocephalus, which is a very rare condition.

**Interventions:**

Mannitol was administered to reduce intracranial pressure.

**Outcomes:**

The patient was discharged from the hospital 30 days after admission, with a final GCS score of 15 and without weaknesses. At follow-up 2 months after discharge, brain CT revealed non-recurrence of hydrocephalus.

**Lessons:**

A blood clot of any size in the ventricle is likely to lead to obstructive hydrocephalus. Prolonged bed rest for IVH patients may help to reduce the incidence of delayed OH.

## 1 Introduction

Cerebral aneurysm (CA) is a common diagnosis in neurosurgical practice, and CA rupture has a high morbidity and mortality rate.^[1]^ A ruptured CA occasionally causes obstructive hydrocephalus (OH), which could lead to severe neurological deficits, including life-threatening brain herniation. This type of OH, which frequently requires external ventricular drainage (EVD), usually occurs early after intracranial hemorrhage due to CA rupture.^[2,3]^ Late-onset hydrocephalus is typically chronic hydrocephalus requiring permanent cerebrospinal fluid (CSF) diversion, in contrast to transient OH (TOH).^[4]^ Therefore, TOH is rare in convalescent patients after CA rupture. Herein, we present a patient with a ruptured CA who experienced TOH because of temporary obstruction of the aqueduct by a blood clot migrating into the fourth ventricle on day 19 after the CA rupture.

## 2 Case presentation

**Figure 1:**
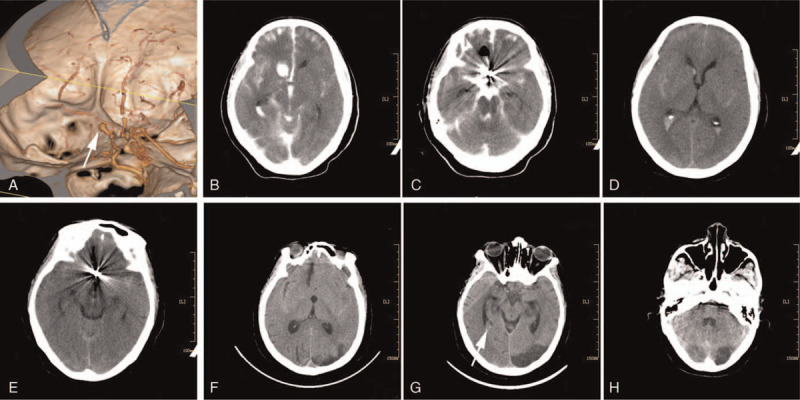
(A) Computed tomographic angiography (CTA) showed an anterior communicating artery aneurysm measuring 3 mm × 5 mm (as indicated by the arrow). (B and C) An urgent computed tomography (CT) after intraoperative bleeding showed subarachnoid hemorrhage (SAH) and intraventricular hemorrhage (IVH) mixed with the leaked contrast agent. (D and E) CT performed on day 6 showed hemorrhage in subarachnoid space, right frontal horn and bilateral occipital horns. (F–H) CT performed on day 12 showed a new delayed cerebral infarction in the left occipital lobe, a blood clot in the right temporal horn (as indicated by the arrow), and a reduction of SAH. No hydrocephalus or clots in the third ventricle (3 V) were noted.

A 64-year-old woman was admitted to the hospital with complaints of dizziness, headache, nausea, and vomiting for 2 hours. Computed tomography (CT) of the brain at admission revealed subarachnoid hemorrhage (SAH). Subsequent CT angiography showed a ruptured anterior communicating artery (AcomA) aneurysm measuring 3 mm × 5 mm (Fig. 1A). She was assessed as Hunt–Hess grade II and had a Glasgow Coma Scale (GCS) score of 15, with no focal deficits. Coil embolization of the aneurysm was performed on day 2 after onset. However, when the second coil was being pushed into the sac, leakage of contrast medium was noticed, indicating further hemorrhage. The leakage subsequently stopped, and the aneurysmal packing density was considered acceptable after the operating physician quickly released the second coil. The patient's pupils were equal at 1.5 mm. An urgent head CT showed SAH and intraventricular hemorrhage (IVH) mixed with the leaked contrast agent (Fig. 1B and C). The patient was admitted to the intensive care unit, and her consciousness returned gradually to being partially responsive (GCS E2M6T) within several hours. To avoid worsening conditions, she was not extubated until her GCS was 13/15 (E3V4M6), the morning after the embolization. The patient was transferred back to the general ward on day 4. A CT scan on day 6 demonstrated hemorrhage in the subarachnoid space, right frontal horn, and bilateral occipital horns (Fig. 1D and E). On day 8, lumbar drainage was performed to drain the CSF contaminated with blood and contrast agent. On day 12, with a GCS of 15, another head CT scan was performed, showing a new delayed cerebral infarction in the left occipital lobe, a blood clot in the temporal horn, and a reduction of SAH. No hydrocephalus or clots in the third ventricle (3 V) were noted (Fig. 1F and H).

**Figure 2:**
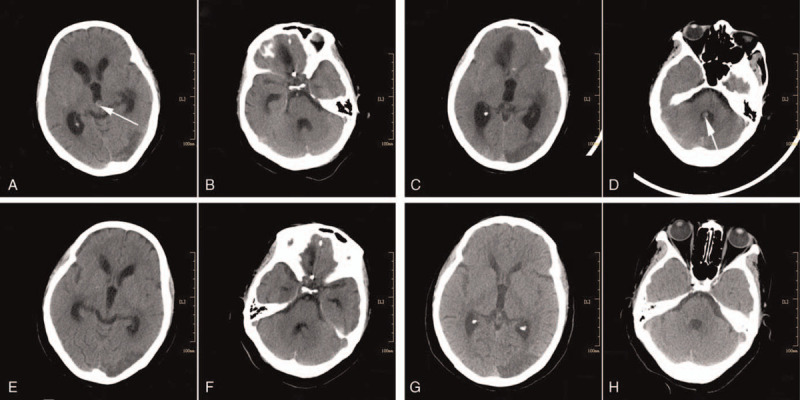
(A and B) Computed tomography (CT) performed at 10:13 am on day 19 showed that there was a blood clot in the third ventricle (3 V, as indicated by the arrow), accompanied by mild hydrocephalus. No clot was seen in the fourth ventricle (4 V). (C and D) CT performed at 2:57 pm on day 19 showed hydrocephalus aggravation, and the previous clot located currently in the 4 V. (E and F) CT performed on day 21 showed remarkable improvement of the hydrocephalus and complete dissolution of the clot in the 4 V. (G and H) At follow-up 2 months after discharge, CT revealed nonrecurrence of hydrocephalus.

However, her condition deteriorated on the morning of day 19. About an hour after standing activities, she complained of a worsening headache and became drowsy. A head CT (performed at 10:13 am) showed that the clot in the right temporal horn had migrated into the 3 V, accompanied by mild hydrocephalus; no clot was seen in the fourth ventricle (4 V) (Fig. 2A and B). Mannitol was administered to reduce intracranial pressure (ICP). However, the patient was found in a coma at about 2:20 pm. An urgent CT (2:57 pm) showed hydrocephalus aggravation, and the previous clot located currently in the 4 V (Fig. 2: C and D). Afterward, the patient had a gradual improvement in consciousness level, awakening at about 6 pm. Owing to the consciousness level improvement, EVD was not performed. Consecutive CTs, therefore, revealed a dynamic process, with clot migration from the right temporal horn into the 4 V through the 3 V and aqueduct. These dynamics suggest that the transient disturbance of consciousness was due to TOH, caused by a small blood clot temporarily blocking the aqueduct. Thereafter, her recovery was uneventful.

A further CT scan performed on day 21 showed remarkable improvement of the hydrocephalus and complete dissolution of the clot in the 4 V (Fig. 2E and F). The patient was discharged from the hospital 30 days after admission, with a final GCS score of 15 and without weaknesses. At follow-up 2 months after discharge, brain CT revealed nonrecurrence of hydrocephalus and an old infarction in the left occipital lobe (Fig. 2G and H).

## 3 Discussion

Acute TOH in the late stage after ruptured CA is extremely rare. To the best of our knowledge, this article describes only the second case reported.^[5]^ Even in acute TOH cases due to other causes of spontaneous intracranial hemorrhage (SIH), there are only sporadic reports^[6–10]^ in the literature. Acute OH frequently occurs in the early stages of SIH. The most common hydrocephalus subtype in the late stages of SIH is communicating hydrocephalus.^[4,11]^ In previous studies regarding TOH following SIH, the most extended time interval between hemorrhagic stroke and TOH was 3 days,^[6]^ in contrast with the 19-day time interval in this report.

Previous studies noted that the reasons for rapid OH remission mainly include the following 2 aspects: acute OH resulting from a clot obstructing the aqueduct resolves when the CSF circulation is restored after clot dissolution^[6–8]^; OH resulting from a clot blocking the aqueduct can cause an increase in ICP.^[9,12]^ The increased ICP will force the clot migration. When the clot reaches the 4 V, the CSF circulation is restored, and the OH resolves. This case report could trace the TOH from occurrence to remission, owing to repeated head CTs capturing a rare dynamic change of a clot migrating from lateral ventricle [LV] to 3 V and 4 V, resulting in a temporary obstruction. This clot migration strongly suggests that the temporary aqueduct blockage was the mechanism causing the TOH. How did the clot migrate? We speculate that the changes in body position (standing activity, as opposed to the previous bedridden position) facilitated the movement from the temporal horn into the body of the LV, following the CSF flow. Then, the clot reached the 3 V through the foramen of Monro, possibly helped by gravity. When the aqueduct inlet, below the 3 V, was obstructed by the migrating clot, blocking the CSF circulation, mild hydrocephalus occurred, followed by headaches and drowsiness. The hydrocephalus gradually aggravated, due to the continued production of CSF, finally leading to a coma. After the clot migration into the 4 V, the hydrocephalus resolved, with the gradual improvement of the patient's consciousness level.

However, not every OH resulting from a small migrating clot can resolve autonomously. Komatsu et al^[13]^ reported a patient with delayed OH due to SIH. The patient received EVD, followed by neuroendoscopic surgery to remove the clot blocking the aqueduct. Therefore, in the presence of a blood clot of any size in the ventricle, the patient should be closely monitored for delayed OH, even if in stable conditions during the recovery period. Although spontaneous remission of the delayed OH is possible in some patients, it is difficult to predict the outcome. If timely treatment cannot be provided to these patients, they may suffer severe neurological consequences, including life-threatening brain hernias. Therefore, we need to consider the late-onset of OH while planning the discharge of IVH patients.

Another concern was the development of a delayed cerebral infarction. This type of delayed cerebral ischemia (DCI) is supposedly caused by cerebral vasospasm after aneurysmal SAH (aSAH). Previous studies suggested that DCI usually occurs between 4 and 14 days following aSAH, indicating that after 14 days, it is rare.^[14,15]^ To the best of our knowledge, the most extended time interval reported between the onset of aSAH and the occurrence of DCI was 21 days.^[16]^ The incidence of DCI-related cerebral infarction is 17%.^[17]^ Based on the CT scan that initially showed the infarction in our patient, it was estimated that the DCI occurred between 6 and 12 days after aSAH onset (Fig. 1F–H). Previous studies reported that lumbar drainage could reduce the incidence of DCI^[18,19]^; however, in this case, it was not sufficient to prevent cerebral infarction.^[20,21]^

## 4 Conclusions

Our case report suggests several considerations: the mechanism of TOH following SIH is a temporary CSF flow obstruction; if a blood clot is not entirely dissolved in patients with IVH, OH may occur and should be considered; bed rest for IVH patients may help to reduce the incidence of delayed OH. Therefore, delayed OH should be considered when planning to discharge patients with IVH.

## Author contributions

**Data curation:** Yuanhong Ge, Wenyu Wang.

**Writing – original draft:** Qingjia Lai.

**Writing – review & editing:** Yuanhong Ge, Xuejun Xu.
